# Turnover in male dominance offsets the positive effect of polygyny on within-group relatedness

**DOI:** 10.1093/beheco/arac121

**Published:** 2023-02-06

**Authors:** Mark Dyble, Tim H Clutton-Brock

**Affiliations:** Department of Anthropology, University College London, 14 Taviton Street, London WC1H 0BW, UK; Department of Zoology, University of Cambridge, Downing Street, Cambridge CB2 3EJ, UK

**Keywords:** kinship, polygyny, relatedness, reproductive skew

## Abstract

Evidence of an association between cooperative breeding systems and average coefficients of relatedness between group members in vertebrates have led to increased interest in the social and ecological factors affecting average kinship within groups. Previous studies have suggested that polygynous mating systems and high degrees of male reproductive skew increase average relatedness because they increase the proportion of offspring born in each group that are paternal siblings. Although this may be the case in semelparous organisms, in many multiparous polygynous animals, intense competition between males shortens the breeding tenure of males and leads to their frequent replacement by competitors which reduces paternal relatedness and average kinship between members of multigenerational groups. Here, we explore the interaction between male reproductive skew and the frequency of turnover in breeding males and its effects on within-group relatedness. Our theoretical model shows that increases in rates of dominance turnover in polygynous systems can offset the positive effect of male skew on relatedness between group members within seasons, showing that polygynous mating systems will not necessarily lead to significant increases in average relatedness, especially in species where there is extensive overlap between generations among group members.

## Teaser

In species with polygynous mating systems, one male mates with several females, resulting in high male reproductive skew and the production of paternal half siblings. This might be expected to increase genetic relatedness within groups. However, reproductive skew introduces intense competition between males which often results in frequent changes in male dominance. This turnover in dominance means that genetic relatedness within multigenerational groups of polygynous animals may not necessarily be much greater than levels under monogamous mating.

## INTRODUCTION

Across vertebrates, the evolution of reproductive cooperation and organizational social complexity is positively associated with genetic relatedness between social partners (Griffin 2003; Hughes et al. 2008; Fisher et al. 2013; Bastiaans et al. 2016; Downing et al. 2020). For example, as inclusive fitness theory would predict (Hamilton 1964, 1971; Gardner et al. 2011), mammalian species showing high rates of cooperative behavior (including alloparental provisioning, a division of labor, and lower rates of competitive behavior) commonly live in groups where there is relatively high average level of kinship between female group members (Lukas and Clutton-Brock 2018).

These findings should focus interest on the processes affecting average kinship between group members. Several important factors affecting average levels of kinship within groups have already been identified (Dyble and Clutton-Brock 2020). Relatedness will typically be lower in larger groups (Wright 1949; Rousset 2004; Lehmann and Rousset 2010). For example, among chimpanzees (*Pan troglodytes*) (Lukas et al. 2005) and lions (*Panthera leo*) (Spong et al. 2002) there is a negative relationship between group size and mean within-group relatedness. For human foraging societies, Walker (2014) estimated that doubling group size from 30 to 60 individuals will more than halve within-group relatedness.

Migration and dispersal patterns also influence relatedness. Higher rates of migration between groups are expected to decrease within-group relatedness (Wright 1949; Queller 1994; Rousset 2004) and sex-specific dispersal patterns can generate sex differences in survival and in the relatedness of males and females to their groupmates, with possible consequences for social behavior (Croft et al. 2021; Ellis et al. 2022). In most group-living mammals, female philopatry is common and usually results in a higher degree of relatedness between co-resident adult females than among adult males (e.g., as seen among lions (Spong et al. 2002) and bottle-nosed dolphins (*Tursiops aduncus*) (Möller and Beheregaray 2004)) though, in a minority of species, including all three African apes, females commonly disperse to other breeding groups after reaching sexual maturity although males may remain and breed in their birth groups so that the usual pattern of sex differences in relatedness is reversed (Clutton-Brock 2016).

Aspects of life history can also influence within-group relatedness. For example, in a recent paper (Dyble and Clutton-Brock 2020), we showed that polytocy (the production of litters of offspring), can increase within-group relatedness because it results in the production of sets of offspring within the same cohort who are maternal siblings, related by at least *r* = 0.25. In the same analysis, we also showed that, as a result of temporal changes in breeding success and survival in resident breeders, within-group relatedness is likely to be lower when the juvenile members of the same group include a relatively high proportion of juveniles from different cohorts, as is common in long-lived species. Protracted lifespans and increasing overlap between generations will also increase variance in the age of individuals and therefore in the extent to which individuals in the same group will share parents.

**Figure 1:**
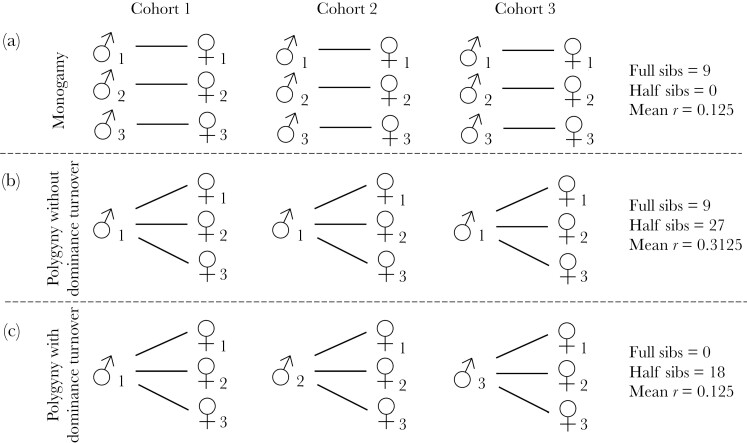
Illustrative example of the effect of male reproductive skew and male dominance turnover on relatedness. In each panel, nine offspring are produced such that there are 36 dyads. Full sibs are related by r = 0.5, half sibs by r = 0.25, and unrelated individuals by r = 0. In panel A, 9 full siblings and no half siblings are produced such that mean relatedness in panel a is (9 * 0.5)/36 = 0.125. In panel B, 9 full siblings and 27 half-siblings are produced such that mean relatedness is ((0.9 * 0.5) + (27*0.25))/36 = 0.3125. In panel C, no full siblings are produced and 18 half-siblings are produced such that mean relatedness is (18*0.25)/36 = 0.125. In panel B, all half sibs are paternal half sibs. In panel C there are nine paternal half sibs and nine maternal half sibs.

Finally, within-group relatedness is influenced by mating systems and by the degree of reproductive skew among female and male breeders (Altmann 1979; Boomsma 2009; Widdig 2013). In previous work (Dyble and Clutton-Brock 2020), we predicted that male and female reproductive skew are key factors in determining within-group relatedness. This is clearest at the extreme—for example, in species with a very high degree of skew within both sexes, mean within-group relatedness can approach, or even exceed, *r *= 0.5, the average relatedness between full siblings (Burland et al. 2002; Randall et al. 2007). Usual levels of reproductive skew in both sexes vary widely between species, though in many social mammals, multiple females breed in each group and females typically show relatively low levels of reproductive skew within years (Clutton-Brock 2016, 2021). In contrast, reproductive skew in males is often relatively high because there are a substantial number of species where a single male virtually monopolizes reproduction in each group and in many species where groups include multiple breeding males, the breeding access of males depends on their social rank, generating large differences in success between individuals (Clutton-Brock 1988; Widdig et al. 2004; Stoinski et al. 2009). As a result, in species that live in groups that include multiple breeding females, variation in male skew is often likely to be one of the principal causes of variation in average kinship between group members (Altmann 1979; Lukas et al. 2005). One principal way in which male reproductive skew affects within-group relatedness is through its effects on increased paternal relatedness among offspring born in the same year and, in a previous model, we demonstrated this effect across a range of life history and group composition parameters and showed that, *all else being equal*, increased male reproductive skew is predicted to increase within-group relatedness (Dyble and Clutton-Brock 2020). For example, if one male can monopolize all reproductive opportunities in a single breeding season then all the offspring born in that cohort will be at least half-siblings. For example, three males mating monogamously with three monotocous females for one breeding season will produce a cohort of unrelated offspring (*r* = 0). In contrast, a single male mating polygynously with three monotocous females for one breeding season will produce a set of half-siblings, related by *r* = 0.25. If that male were to exclusively mate with the three females for three seasons, relatedness across the three cohorts of offspring would be 2.5 times greater than under monogamous mating (Figure 1A and B, *r* = 0.3125 under polygyny vs *r *= 0.125 under monogamy).

**Figure 2:**
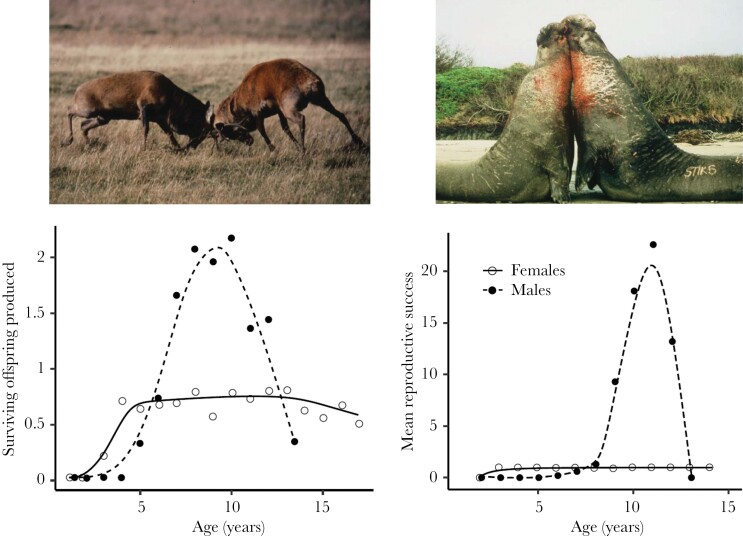
Reproductive success by age and sex for red deer (left) and elephant seals (right). Based on Clutton-Brock (2021). Original data from Boeuf and Reiter (1988) and Clutton-Brock et al. (1988). Photographs: (left) Tim Clutton-Brock, (right) Burney Le Bouef.

In reality, however, all else is not equal because in many systems polygynous mating is associated with intense competition between males for access to females with the result that the reproductive dominance of males is often short-lived and the reproductive tenure of individual males declines as the degree of polygyny and the extent of male reproductive skew increases (Lukas and Clutton-Brock 2014; Clutton-Brock 2021). For example, in many strongly polygynous species like red deer (*Cervus elaphus*) and elephant seal (*Mirounga sp.*) where successful males may breed with more than 10 females in a single season, males rarely maintain their dominance for more than a few years whereas female breeding lifespans may be substantially longer ([Boeuf and Reiter 1988; Clutton-Brock et al. 1988], Figure 2). In monotocous species, successive dominant males are seldom closely related to each other, so that high rates of male turnover offset the effects of male reproductive skew on group relatedness and, in extreme cases, may negate them entirely. For example, if three females mate polygynously with a different male in three successive seasons, the resulting offspring will be related by *r* = 0.125 on average (Figure 1C) and kinship between their offspring will be the same as expected under monogamous mating (*r* = 0.125, though note that the nine full sibs are replaced by 18 half sibs).

Previous work has considered the effect of turnover in male dominance on the proportions of paternal and maternal half-siblings produced within and between reproductive cohorts (Altmann 1979; Widdig 2013) and of the effect of male reproductive skew on group relatedness (Lukas et al. 2005). Here, we expand on this and on our previous model (Dyble and Clutton-Brock 2020) to explore the combined effects of male reproductive skew and turnover in male dominance tenure on average within-group relatedness. We show that the short male reproductive tenures commonly associated with high male skew have the potential to offset the positive effect of male skew on within-group relatedness.

## METHODS

It is important to note that we are considering male reproductive skew as the distribution of the share of paternity across several males over a relatively short period of time such as a breeding season or year. This is how male reproductive skew is commonly conceptualized and measured and is different from variation in lifetime reproductive success (Clutton-Brock 1988; Dugdale et al. 2008). This is an important point because high male reproductive skew in the short term can still lead to relatively low levels of variation in male lifetime reproductive success if male dominance tenure is very short (Lukas and Clutton-Brock 2014).

To explore the relationship between male reproductive skew, male tenure, and mean intragroup relatedness, we adapted our existing mathematical model of within-group relatedness (Dyble and Clutton-Brock 2020). Although this model makes simplifying assumptions about how dispersal, mating, and demography work, it is nonetheless able to accurately predict observed variation in within-group relatedness across a sample of mammals (see Dyble and Clutton-Brock 2020). Our original model had eleven parameters: the number of males (*N*_*m*_), number of females (*N*_*f*_), number of juvenile cohorts in the group at the same time (*n*), male reproductive skew (*α*) probability of subordinate female reproduction (*β*), litter size (k), male dominance tenure (*τ*_*m*_), female dominance tenure (*τ*_*f*_), the number of juveniles per adult (*θ*), male dispersal, and female dispersal. Although female reproductive skew can have important consequences for social behavior, our focus here is on polygyny and therefore male reproductive skew. Because polygyny assumes a plural breeding system in which multiple females breed, we simplify the model by holding the probability of subordinate female reproduction (*β*) at 1 such that all females breed with equal success. In doing so, the female tenure parameter (*τ*_*f*_) becomes redundant. The model considers relatedness in a group with *n* juvenile cohorts. For seasonal breeders, a cohort is the set of juveniles born in a season. For year-round breeders, the cohorts in our model represent the set of juveniles born within a single inter-birth interval period such that a female will give birth no more than once within that period.

The key parameters in the present analysis are male reproductive tenure (*τ*_*m*_) and male reproductive skew (*α*). *τ*_*m*_ determines the probability that the dominant male in the group maintains his reproductive dominance from one cohort of juveniles to the next. If he does not (with probability 1- *τ*_*m*_), a random male is chosen as the new dominant. When *τ*_*m *_= 0, there is turnover in male dominance between each cohort and when *τ*_*m *_= 1, the dominant male never loses his dominance. *α* determines the proportion of the reproductive success of other males that is taken by the dominant male. Specifically, the total share of reproduction taken by the dominant male (*d*) is 1/*N*_*m*_ + *α*(*N*_*m*_ − 1)/*N*_*m*_. As such, when *α* = 0, all males including the dominant have an equal probability of fathering an offspring (1/*N*_*m*_), whereas when *α* = 1, the dominant male sires all offspring in that cohort. We refer to *α* as male reproductive skew because this parameter determines the degree of skew in the group. However, it should be noted that *α* is not a measure of reproductive skew as used in empirical studies, of which many exist, (see Kokko et al. 1999; Nonacs 2000; Ross et al. 2020) although *α*^*2*^ is equal to Bradbury’s bounded skew index (Bradbury et al. 1985).

Given *d*, the probability of individuals from the same cohort sharing a father (*p*_s_) is:


$$\begin{array}{l} p_{s}=d^{2}+{\left(1-d\right)}^{2}/\left(N_{m}-1\right) \end{array}$$

and the probability of individuals from different cohorts sharing a father (*p*_*d*_) is:


$$\begin{array}{l} p_{d}=\;\frac{\sum_{i}^{n-1}2(n-i)\left({\tau_{m}}^{i}p_{s}+\left(\frac{1-{\tau_{m}}^{i}}{N_{m}}\right)\right)}{n^{2}-n} \end{array}$$

Given the above, the probability of two juveniles sharing a father (*P*) is:


$$\begin{array}{l} P=\;\frac{np_{s}+\;(n^{2}-n)p_{d}}{n^{2}} \end{array}$$

With the removal of female dominance, the probability of two juveniles sharing a mother (*M*) is simplified from the original model to (*nk—*1)/*nkN*_*f*_, where *nk*–1 is the expected number of maternal siblings across all cohorts and *nkN*_*f*_ is the total number of offspring born (*n* is the number of juvenile cohorts in the group, *k* is the litter size, and *N*_*f*_ the number of females). Given *P* and *M,* the relatedness through only parental generation is 0.25(*P *+ *M*). Given an assumption of female philopatry (the typical mammalian pattern), we then incorporate additional relatedness between juveniles owing to matrilineal relatedness at the grandparental generation or deeper (up to four generations), estimating relatedness among juveniles as:


$$\begin{array}{l} r_{j}=\;\frac{P+M}{4}\;\underset{i=1}{\overset{4}{\sum }}\;{\left(\frac{1-M}{4}\right)}^{\left(i-1\right)} \end{array}$$

We can then estimate relatedness among adults (*r*_*A*_) and between adults and juveniles (*r*_*B*_) and then combine this to estimate overall within-group relatedness according to the formulae in Dyble and Clutton-Brock (2020) and given here in the Supplementary Material.

In order to assess the effect of male skew and tenure on relatedness we first set *N*_*m*_, *N*_*f*_, *n*, *k*, θ, and dispersal to fixed at typical mammalian values (see Table 1) and calculated mean within-group relatedness under varying degrees of turnover in male dominance tenure (*α*) and male reproductive skew (*τ*_*m*_). To explore the generalizability of these results across plausible mammalian social systems, we then ran 10^5^ iterations in which we randomly set all model parameters within the ranges listed in Table 1 except for *τ*_*m*_ and *α*. In each iteration, we calculated the predicted within-group relatedness given these random parameter values and two different values for *τ*_*m*_ (*τ*_*m*_ = 0 and *τ*_*m*_ = 1) and two different values for *α* (*α* = 0 and *α* = 1). Doing so allows us to estimate how much relatedness will increase when the degree of male skew changes from no skew (*α* = 0) to complete reproductive dominance by the dominant male (*α* = 1), under conditions of both high turnover in male dominance (*τ*_*m*_ = 0) and no turnover in male dominance (*τ*_*m*_ = 1). Finally, we ran an additional set of 10^6^ iterations in which we also randomly set τ_m_ as well as *N*_*m*_, *N*_*f*_, *n*, *k*, and *θ* and estimated relatedness under *α* = 0 and *α* = 1. This allows us to explore the conditions under which increased male skew will have a substantial effect on within-group relatedness. It is worth noting that although each of the parameters is set independently in our randomization process, many of these social and life history parameters co-vary in reality. For example, male reproductive skew is usually constrained in larger groups because more males increase competition for mates and more females in the group reduce the potential for male monopolization.

**Table 1:** Social and life history parameters explored

Parameter	Symbol	Default values	Range explored
Male reproductive skew	*α*	–	{α ∈ *R* | 0 ≤ α ≤ 1}
Male dominance tenure	*τ*_*m*_	–	{*τ*_*m*_ ∈ *R* | 0 ≤ *τ*_*m*_ ≤ 1}
Number of males	*N*_*m*_	8	{*N*_*m*_ ∈ *Z* | 2 ≤ *N*_*m*_ ≤ 10}
Number of females	*N*_*f*_	8	{*N*_*f*_ ∈ *Z* | 2 ≤ *N*_*f*_ ≤ 10}
Number of juvenile cohorts	*n*	5	{*n* ∈ *Z* | 1 ≤ *n* ≤ 6}
Litter size	*k*	1	{*k* ∈ *Z* | 1 ≤ *k* ≤ 5}
Juveniles per adult	θ	1	{θ ∈ *R* | 0.5 ≤ θ ≤ 2}

## RESULTS

### The effect of male skew on within-group relatedness is diminished by short dominance tenure

**Figure 3:**
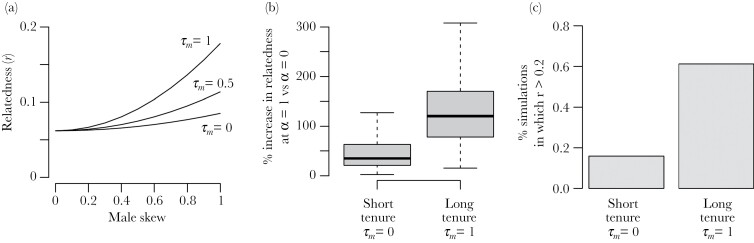
The effect of male skew and tenure on predicted within-group relatedness. (A) estimated within-group relatedness given varying degrees of male skew (*α*) under high turnover in male dominance (*τ*_*m*_ = 0, lower), intermediate turnover in male dominance (*τ*_*m*_ = 0.5, middle), and no turnover in male dominance (*τ*_*m*_ = 1, upper). (B) estimated proportional increase in predicted relatedness resulting from an increase in male skew from no skew (*α* = 0) to full reproductive dominance by the dominant male (*α* = 1), given high turnover in male dominance (*τ*_*m*_ = 0, left) and no turnover in male dominance (*τ*_*m*_ = 1, right). (C) proportion of simulations in which mean relatedness was r > 0.2 under *α* = 1 and *τ*_*m*_ = 0 (left) and *α* = 1 and *τ*_*m*_ = 1 (right); r = 0.2 is somewhat arbitrary but chosen as a within-group relatedness value beyond which the occurrence of organizational social complexity is common in mammals (Lukas and Clutton-Brock 2018).

All else being equal, increased male reproductive skew is predicted to increase within-group genetic relatedness. However, our results show that high turnover in male reproductive tenure can offset the positive effect on male skew on within-group relatedness. For example, in the conditions modeled in Figure 3A (*N*_*m*_ = 8, *N*_*f*_ = 8, *n* = 5, *k* = 1, θ = 1, female philopatry), a change in male skew from *α* = 0 (no male reproductive skew) to *α* = 1 (where all females mate with the same male) results in an almost three-fold increase in relatedness if there is no turnover in male dominance (i.e., *τ*_*m*_ = 1) (increase from *r* = 0.063 to 0.178). However, if there is high turnover in male dominance (*τ*_*m*_ = 0) the increase in within-group relatedness is much more modest (37% increase, from *r* = 0.062 to 0.085).

Across the broad range of parameter values given in Table 1, the proportional increase in within-group relatedness associated with a change in male reproduction skew from a *α* = 0 to *α* = 1 is substantially lower when male tenures are short (*τ*_*m*_ = 0, mean increase = 54%, SD = 53%) compared with when male reproductive tenure is long (*τ*_*m*_ = 1, mean increase = 127%, SD = 61%; Figure 3B). The magnitude of this difference is substantial; although polygyny with long reproductive tenures may increase within-group relatedness in a plural breeding group to values commonly associated with the evolution of highly cooperative societies (Lukas and Clutton-Brock 2018), polygyny with short reproductive tenures is unlikely to do so. For example, 61% of simulations in which *α* = 1 and *τ*_*m*_ = 1 resulted in an estimated relatedness of r > 0.2, compared with 16% of simulations in which *α* = 1 and *τ*_*m*_ = 0 (Figure 3C).

### When is the effect of skew on relatedness most pronounced?

**Figure 4:**
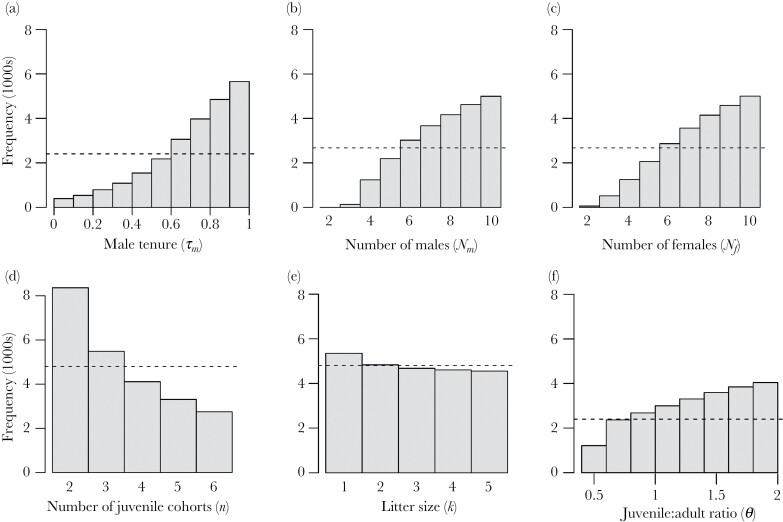
Conditions under which high male reproductive skew leads to a major increase in within-group relatedness. We ran 10^6^ iterations of the model and, in each iteration, randomly set values for all parameters (except *α*) within the ranges listed in Table 1, calculating relatedness given *α* = 0 and *α* = 1 where *α* determines the degree of male reproductive skew. The histograms represent the parameter values under which within-group relatedness more than doubled as a result of this increase in *α* from 0 to 1. The dotted lines represent the expected distribution if the parameter had no effect on change in relatedness. Panels correspond to (A) male dominance tenure; *τ*_*m*_, (B) number of males; *N*_*m*_, (C) number of females; *N*_*f*_, (D), number of juvenile cohorts; *n*, (E) number of juveniles per adult; *θ*, and (F) litter size; *k*.

We ran a final set of 10^6^ iterations in which we also randomly set all model parameters except *α* and estimated relatedness under *α* = 0 and *α* = 1. On average, this change in *α* increased relatedness by 71% (SD = 49%) and relatedness more than doubled in 240,229 of these 10^6^ iterations. Looking at the distribution of parameter values across these 240,229 iterations provides an indication of the conditions under which polygynous mating is most likely to have a large positive effect on within-group relatedness. As expected given the above results, male tenure (τ_m_) had a major influence on group relatedness (Figure 4A). In addition, relatedness increased most dramatically when the number of males (*N*_*m*_) and females (*N*_*f*_) in the group was large (Figure 4B and C), when there are fewer concurrent juvenile cohorts in the population (*n*, Figure 4d), and when there were more juveniles relative to adults in the group (*θ*, Figure 4F). Litter size had little influence on whether or not increased skew led to higher relatedness (*k*, Figure 4E).

## DISCUSSION

All else being equal, increased male reproductive skew is expected to increase within-group relatedness by increasing paternal sibships (Altmann 1979; Lukas et al. 2005; Widdig 2013). However, in many species, high reproductive skew co-occurs with intense competition between males with the result that male reproductive tenure is often short-lived (Lukas and Clutton-Brock 2014). Our results show that high levels of male dominance turnover can offset the positive effect of increased male reproductive skew on within-group relatedness and that the extent of this offset effect is likely to be greatest in long-lived species in which there are multiple juvenile cohorts.

It is worth noting that our model assumes a simple form of reproductive skew whereby one male sires a disproportionate share of offspring born in the cohort and all other males share the remaining paternity equally. This may be a reasonable approximation of how reproductive skew operates in many mammal societies, particularly cases where a single dominant male defends a group of females and other males rely on alternative reproductive strategies to gain mating opportunities. However, this is not always the case and the relationship between dominance and reproductive skew varies between groups and between populations (Clutton-Brock 1998; Johnstone 2000; Port and Kappeler 2010; Duncan 2022). For example, in some species, like lions, coalitions of males defend a group of females, and paternity is shared within the coalition, so that reproductive skew is reduced (Packer et al. 1988, noting that the distribution of paternity between the coalition males can vary). Similarly, among gelada baboons (*Theropithecus gelada*), dominant males will sometimes allow a “follower male” to join and help defend a breeding group. The follower male gains occasional mating opportunities, leading to reductions in annual skew, but their presence in the group extends the dominant male’s tenure by an average 30%, offsetting the costs of their presence to the dominant male (Snyder-Mackler et al. 2012). It is also worth noting that the male with the largest share of paternity is not always the most socially dominant male within the group (Ellis 1995; Engh 2002). Our model also assumes that the membership of the group is unaffected by a change in male dominance so that successful challengers acquire all females held by the previous dominant male. Again, this situation is not always seen. For example, in some species where females disperse to breed, like zebras or hamadryas baboons, social bonds between co-resident breeding females are weak and groups disband after the loss of the breeding male, so that successful challengers do not necessarily acquire the females defended by the previous dominant male and the benefits of attempting to displace resident males are likely to be reduced (Rubenstein and Nuñez 2009; Swedell et al. 2011). It is also worth noting that the effects described in this paper are most likely to occur where male breeding success depends directly on successful competition with other males and that many other factors can influence the degree of skew including female mating preferences.

What are the implications of our results for female skew and within-group relatedness? Mathematically, our results apply equally to male and female reproductive skew; the effect of high female reproductive skew on group relatedness will be diminished by high turnover in female dominance tenure. In reality, however, this is of less importance because female reproductive skew remains relatively low in the majority of mammal societies and in species with high female skew dominance tenures often tend to be relatively long lasting and to be longer than male dominance tenures (Clutton-Brock 2016). For example, among mammals with unusually high levels of reproductive skew in females, like naked mole-rats (*Heterocephalus glaber*) and meerkats (*Suricata suricatta*), the breeding tenure of females is still substantially longer than that of males (Bennett and Faulkes 2000; Clutton-Brock et al. 2006; Duncan 2022). Few studies have yet considered why this may be the case but there appear to be at least two possible explanations. First, female commitments to various forms of parental investment may constrain selection for expensive forms of aggression and competition in females. Alternatively, in species where females usually remain in their birth groups whereas males disperse, it is often the case that all group members are intolerant of unrelated intruding females so that resident dominant females are protected from external usurpers although intruding males can attack and evict resident male breeders and are usually then tolerated by resident females and natal males (Duncan 2022). The potential effects of dispersal patterns on sex differences in tenure are well illustrated in a recent study of tenure in meerkats (*Suricata suricatta*), where a single dominant breeding pair produce most of the surviving offspring in each group and breeding females are mostly natal animals that have not dispersed from their birth group whereas dominant breeding males are immigrants (Clutton-Brock et al. 2006; Clutton-Brock and Manser 2016; Duncan 2022). Here, dominant breeding males have shorter tenure than dominant breeding females because breeding males are more likely than breeding females to be displaced by males from other groups and are also more likely to leave their group voluntarily. As a result, their breeding tenure and the extent of individual variance in lifetime breeding success is reduced in breeding males compared with breeding females (Duncan 2022).

We hope that our results will stimulate investigation of the factors affecting the duration of dominance tenure and breeding lifespans in both sexes in a wider range of species. At the moment, few studies of natural populations of vertebrates have measured individual variance in tenure duration and reproductive skew across the lifespan in both sexes and almost all of those that have done so have focused on sexually dimorphic species with polygynous mating systems (Clutton-Brock 1988; Lukas and Clutton-Brock 2014). As a result, many questions about the inter-relationships between mating systems, male tenure, and male skew remain unanswered. For example, are male tenures longer and sex differences in tenure length smaller in monogamous, pair-living species than in polygynous species? Are male tenures longer and sex differences in breeding tenure smaller in polygynous species that show little sexual size dimorphism, like zebras and some other social equids, where males fight by biting rather than by pushing contests that favor the heaviest contestants? Are male dominance or breeding tenures longer in multi-male species where males rely partly on alliances and coalitions to retain their status than in single-male species where male tenure depends on the size and strength of individual males as some comparative analyses suggest? Is the duration of male tenure shorter in species where females are philopatric and usually remain together after one breeding male displaces another than in species where females habitually disperse to breed and co-resident females usually disperse if the resident breeding male dies or is displaced, as in some social mammals and many birds—so that the reproductive gains of successful challengers are reduced? Finally, how do sex differences in patterns of dispersal affect breeding tenure and variance in breeding success in males and females?

Although our model was produced primarily with vertebrates in mind, our results are also relevant to studies of polygynous invertebrates. In a recent review, Griffin et al. (2019) identified ten insect taxa that exhibit harem polygyny where a single male defends an aggregation of two or more females. Across these species, which include bark beetles (Kirkendall 1983) and angel insects (Choel 1994), we see some of the common features associated with polygyny in mammals such as sexual dimorphism and maternal care (Griffin et al. 2019). Further research is required to assess whether the strong negative relationship between male skew and male tenure seen in mammals (Lukas and Clutton-Brock 2014) is also seen in these species. If it is, then shorter lifespans of these species mean that the timescale of dominance tenure and turnover is likely to be much accelerated for, in some polygynous insects, harems may only last a day or two (Weissmann 1997; Griffin et al. 2019). Given the relationship between relatedness and male reproductive skew and tenure explored here, it would also be of interest to establish the extent to which sociality in these species—and across taxa more broadly—is enhanced or limited by the magnitude of male reproductive skew and the longevity of male reproductive dominance.

Our results may also have implications for understanding genetic diversity within polygynous populations. Theory predicts that an increase in male reproductive skew will reduce effective population size and the degree of genetic diversity within a population (Chesser 1991; Nunney 1993). Just as our results show that turnover in male dominance will offset the positive effect of male skew on within-group relatedness, we also expect dominance turnover to offset the negative effect of male reproductive skew on genetic diversity. This, in addition to the possibility that polygynous mating may maintain genetic diversity through sexual selection favoring heterozygous males (Pérez-González et al. 2009) may mean that genetic diversity in polygynous populations is not as low as might be expected when only considering the degree of male reproductive skew within a single breeding season. Given the importance of genetic diversity and effective population size (*N*_*e*_) in conservation biology (Nunney and Elam 1994; Creel 1998; Keller 2002; Frankham 2005), it may be important that studies estimating genetic diversity from demographic data consider the rate of turnover in male dominance as well as the degree of male reproductive skew at any one point in time.

## Supplementary Material

arac121_suppl_Supplementary_Material_1

arac121_suppl_Supplementary_Material_2
