# Supplementary material for: Turnover in male dominance offsets the positive effect of polygyny on within-group relatedness
Source: Behav Ecol. 2023 Feb 6;34(2):261–8. doi: 10.1093/beheco/arac121 (PMC10047635; doi:10.1093/beheco/arac121)
Supplement: arac121_suppl_Supplementary_Material_1 [file arac121_suppl_supplementary_material_1.pdf]

Turnover in male dominance offsets the positive effect of polygyny on within-group relatedness

Mark Dyble and Tim H. Clutton-Brock

Supplementary Material

Estimating within-group relatedness

Given r_j as provided in the main text and assuming female philopatry, within-group relatedness is estimated first by calculating relatedness among adults which, as in Dyble and Clutton-Brock (2020) is:

$$r_A = \frac{r_J\ ((N_m{}^2 - N_m) + (Nf^2 - N_f))}{\left(N_m + N_f\right)^2 - (N_m + N_f)}$$

We then calculate relatedness between adults and juveniles as:

$$r_B = \frac{1 + 0.5r_J\left(N_f - 1\right) + \ 0.5r_J(N_m - 1)}{N_f + N_m}$$

Using the above, and where N_A is the number of adults ($N_f + N_m$) and N_J is the number of juveniles ($N_J = \theta N_A$), we estimate relatedness across the whole group as:

$$r_G = \frac{r_A N_A(N_A - 1) + \ 2r_B N_A N_J \ + \ r_J N_J\left(N_J - 1\right)}{\left(N_J + N_A\right)\left(N_J + N_A - 1\right)}$$
